# Clinical determinants of plasma cardiac biomarkers in patients with stable chest pain

**DOI:** 10.1136/heartjnl-2019-314892

**Published:** 2019-06-01

**Authors:** Rong Bing, James Henderson, Amanda Hunter, Michelle C Williams, Alastair J Moss, Anoop S V Shah, David A McAllister, Marc R Dweck, David E Newby, Nicholas L Mills, Philip D Adamson

**Affiliations:** 1 BHF Centre for Cardiovascular Science, University of Edinburgh, Edinburgh, UK; 2 Edinburgh Imaging, University of Edinburgh, Edinburgh, UK; 3 Institute of Health and Wellbeing, University of Glasgow, Glasgow, UK; 4 Usher Institute of Population Health Sciences and Informatics, University of Edinburgh, Edinburgh, UK; 5 Christchurch Heart Institute, University of Otago, Christchurch, New Zealand

**Keywords:** computed tomography coronary angiography, troponin, B-type natriuretic peptide, cardiac biomarkers, coronary artery disease

## Abstract

**Objective:**

Troponin and B-type natriuretic peptide (BNP) concentrations are associated with cardiovascular risk in stable patients. Understanding their determinants and identifying modifiable clinical targets may improve outcomes. We aimed to establish clinical and cardiac determinants of these biomarkers.

**Methods:**

This was a prespecified substudy from the randomised Scottish Computed Tomography of the Heart trial, which enrolled patients 18–75 years with suspected stable angina between 2010 and 2014 (NCT01149590). We included patients from six centres in whom high-sensitivity troponin I and BNP were measured (Singulex Erenna). Patients with troponin >99th centile upper reference limit (10.2 ng/L) or BNP ≥400 ng/L were excluded to avoid inclusion of patients with myocardial injury or heart failure. Multivariable linear regression models were constructed with troponin and BNP as dependent variables.

**Results:**

In total, 885 patients were included; 881 (99%) and 847 (96%) had troponin and BNP concentrations above the limit of detection, respectively. Participants had a slight male preponderance (n=513; 56.1%), and the median age was 59.0 (IQR 51.0–65.0) years. The median troponin and BNP concentrations were 1.4 (IQR 0.90–2.1) ng/L and 29.1 (IQR 14.0–54.0) ng/L, respectively. Age and atherosclerotic burden were independent predictors of both biomarkers. Male sex, left ventricular mass and systolic blood pressure were independent predictors of increased troponin. In contrast, female sex and left ventricular volume were independent predictors of increased BNP.

**Conclusions:**

Troponin and BNP are associated with coronary atherosclerosis but have important sex differences and distinct and contrasting associations with CT-determined left ventricular mass and volume.

**Clinical Trial registration:**

NCT01149590; Post-results.

## Introduction

The diagnostic and prognostic application of myocardial-specific plasma proteins is widely accepted in current practice. Two such cardiovascular biomarkers are high-sensitivity cardiac troponin and B-type natriuretic peptide (BNP). Although developed for use in specific populations, both are recognised to be important indicators of adverse prognosis among stable patients, even in the absence of established cardiac disease.^1–4^ These associations likely reflect the role of myocardial proteins as surrogate measures of underlying processes such as hypertension, atherosclerosis or left ventricular dysfunction.^5 6^ Indeed, there have been suggestions that measurement of high-sensitivity troponin and BNP in certain asymptomatic populations may be of benefit.^7^ However, these tests are not specific for a single disease process. Understanding the drivers of biomarker concentrations in an individual patient is therefore crucial to facilitate interpretation of results and guide management.

Cardiac CT is a valuable investigation that is widely used for the diagnosis of coronary artery disease. However, it can also provide an assessment of left ventricular mass and volume, both of which are associated with elevated biomarker concentrations.^8 9^ How these measures contribute to variations in biomarker concentrations in stable patients remains unclear.

In this biomarker substudy of the Scottish Computed Tomography of the Heart (SCOT-HEART) trial, we aimed to establish the clinical and cardiac determinants of plasma high-sensitivity cardiac troponin I and BNP concentrations in patients presenting with stable chest pain. We hypothesised that, in addition to recognised clinical determinants, ventricular mass and volume as determined by cardiac CT would be associated with higher biomarker concentrations.

## Methods

### Study design and population

This is a post-hoc analysis of the open-label, randomised SCOT-HEART trial. The trial design, primary analysis and 5-year outcomes have been published.^10–12^ Patients 18–75 years of age referred by a primary care physician to a cardiology clinic with stable chest pain were enrolled after obtaining written informed consent from 12 cardiology centres across Scotland. In total, 4146 patients were recruited from November 2010 to September 2014. Patients with severe chronic kidney disease (serum creatinine >200 μmol/L or estimated glomerular filtration rate <30 mL/min/1.73 m^2^) or acute coronary syndrome within 3 months were excluded. All patients underwent routine clinical evaluation. A clinical diagnosis and management plan were documented prior to recruitment. Eligible patients were randomised 1:1 to receive routine care or routine care plus CT coronary angiography (CTCA).

For this substudy, we included patients who had been referred from six centres for their allocated CTCA in Edinburgh (n=1317). As this study aimed to characterise the determinants of baseline cardiac biomarkers in stable patients without acute myocardial injury or heart failure, patients with troponin concentrations >99th centile upper reference limit (10.2 ng/L) (n=29) or BNP ≥400 ng/L (n=11)^13^ were excluded.

### Computed tomography coronary angiography

The methods used to perform and to analyse coronary findings from CTCA have been reported.^12^ Intraobserver and interobserver agreement were excellent (95% and 91%, respectively).^10^ Atherosclerotic burden was quantified according to the CT-adapted Leaman score. This validated tool provides a score based on plaque location, composition (non-calcified, calcified or mixed) and degree of stenosis (<50% or ≥50%). The score has been validated in several cohorts; a cut-off of >5 provides additive prognostic value beyond stenosis alone.^14^

### Left ventricular mass and volume

Cardiac CT assessments of myocardial mass and volume are validated and readily performed. Older protocols used retrospective electrocardiographic gating; however, prospective gating is now routine, with image acquisition performed in mid-diastole. Although this limits the available phases, mid-diastolic left ventricular mass measurements correlate well with other phases and have prognostic value.^15^ Left ventricular mid-diastolic volume measurements also correlate with end-diastolic volumes and have prognostic importance,^16^ with established thresholds for normal ranges.^17 18^ Due to variation in volume measurements across the cardiac cycle, we only included scans with gated images available between 70% and 90% of the R-R interval for this metric. In contrast, estimation of left ventricular mass is less affected by the cardiac cycle; we therefore included scans with images between 50% and 90% of the R-R interval.

Using automated software (Vitrea, Vital Images, Minnetonka, USA), left ventricular myocardial contours were detected during mid-diastole (75% phase or nearest acquired) in two planes and 0.7 mm slices and manually adjusted to ensure accurate borders, allowing calculation of left ventricular volume and myocardial volume (from which myocardial mass is derived). Corrections for mid-diastolic acquisition were then applied^15 19^ to provide mass and estimated left ventricular end-diastolic volume. Values were indexed to body surface area (Du Bois formula). Left ventricular hypertrophy was defined as indexed left ventricular mass >79.2 g/m^2^ for men and >63.8 g/m^2^ for women—CT-specific thresholds with prognostic value.^15^

### Biomarker measurement

Troponin was measured using the Erenna high-sensitivity cardiac troponin I assay (Singulex, Alameda, California). The limit of detection is 0.1 ng/L with a 99th centile upper reference limit of 10.2 ng/L and a limit of quantification (LOQ; coefficient of variation <10%) of 0.4 ng/L.^20 21^ The BNP assay (Singulex) had a limit of detection of 2.0 ng/L and LOQ (coefficient of variation <10%) of 3.4 ng/L. EDTA-treated plasma samples were stored at −80°C.

### Statistical analysis

Analyses were performed using biomarker concentrations as continuous variables following log_2_ transformation. For descriptive purposes baseline characteristics are reported as median (IQR) according to troponin and BNP quantiles. Body mass index, systolic blood pressure, diastolic blood pressure, CT-adapted Leaman score, indexed left ventricular mass, indexed left ventricular volume and creatinine were log-transformed as a linearising transformation. Missingness was low, apart from creatinine (n=220, 25%) and indexed left ventricular volume (n=195, 22%; due to available scan phases and image quality). To avoid introducing bias by using complete case analysis, missing values were estimated by multivariable imputation by chained equations. Subsequent analyses were conducted using a single imputed value for each missing value. In a sensitivity analysis, the pooled parameters for the final models were also determined (online supplementary appendix).^22^ Patients with troponin or BNP concentrations below the lower limit of detection (n=4 and n=38, respectively) were assigned a value of half the lower limit of detection. Univariable and multivariable linear regression modelling were performed to identify the associations between each biomarker and relevant clinical, biochemical and imaging-derived variables. Included variables were CT-adapted Leaman score, left ventricular mass and volume indexed to body surface area, age (per decade), male sex, hypertension, hyperlipidaemia, diabetes, family history of coronary heart disease, documented coronary heart disease, smoking history, systolic blood pressure, diastolic blood pressure, body mass index, and serum creatinine. Sequential multivariable models were built, beginning with CT-adapted Leaman score and indexed left ventricular mass and volume, followed by the addition of clinical history, clinical measurements and biochemistry (online supplementary tables 1 and 2). Two-sided p values <0.05 were considered statistically significant. Analysis was performed using R V.3.5.0 (R Foundation for Statistical Computing, Vienna, Austria).

10.1136/heartjnl-2019-314892.supp1 Supplementary file 1

## Results

### Patient characteristics

**Figure 1:**
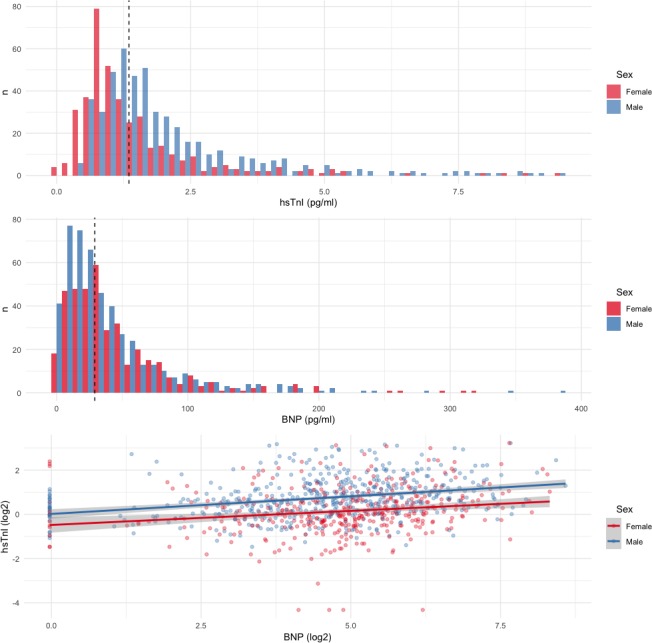
Distribution of cardiac biomarkers between men and women. Panels A and B demonstrate the distribution of high-sensitivity troponin I (hsTnI) and B-type natriuretic peptide (BNP), stratified by gender. Dashed lines represent median values for hsTnI (1.4 ng/L) and BNP (29.1 ng/L) for the whole cohort. Panel C represents univariable linear regression modelling that demonstrates the weak association between hsTnI and BNP for both women (r^2^=0.03, coefficient 0.13, p<0.001) and men (r^2^=0.09, coefficient 0.16, p<0.001).

In total, 943 patients underwent biomarker sampling, of whom 29 (3%) had a troponin concentration >99th centile upper reference limit and 11 (1%) had a BNP >400 ng/L. After excluding these patients and patients with missing BNP data, 885 patients were included. The median troponin concentration was 1.4 (0.9–2.1) ng/L. The median BNP concentration was 29.1 (14.0–54.0) ng/L (figure 1). Patients with higher biomarker concentrations were older, more likely to have a history of coronary heart disease, be receiving medical therapies for cardiovascular conditions and have a typical history of angina (table 1). Troponin concentrations were higher in men than in women (1.6 (1.1–2.1) vs 1.0 (0.7–1.6) ng/L) in contrast to BNP concentrations which were higher in women than in men (31.9 (17.7–58.1) vs 26.5 (12.5–50.0) ng/L). There was a weak correlation between troponin and BNP concentrations (Pearson’s r=0.21, p<0.001).

**Table 1:** Baseline characteristics for overall cohort and according to high-sensitivity cardiac troponin I and BNP quantiles (below/above median)

	Overall N=885	hs-cTnI			BNP		
		Low (≤1.4 ng/L) n=443	High (>1.4 ng/L) n=442	P value	Low (≤29.1 ng/L) n=443	High (>29.1 ng/L) n=442	P value
Male	494 (55.8)	192 (43.3)	302 (68.3)	<0.001	267 (60.3)	227 (51.4)	0.009
Age	59.0 (51.0–65.0)	55.0 (48.0–63.0)	62.0 (55.0–67.0)	<0.001	55.0 (48.0–62.0)	62.0 (55.0–67.0)	<0.001
Hypertension	325 (36.7)	119 (26.9)	206 (46.6)	<0.001	149 (33.6)	176 (39.8)	0.07
Hyperlipidaemia	528 (59.7)	246 (55.5)	282 (63.8)	0.015	233 (52.6)	295 (66.7)	<0.001
Diabetes	91 (10.3)	41 (9.3)	50 (11.3)	0.37	50 (11.3)	41 (9.3)	0.38
Known CAD	77 (8.7)	24 (5.4)	53 (12.0)	0.001	23 (5.2)	54 (12.2)	<0.001
Family history of CAD	383 (43.3)	214 (48.3)	169 (38.2)	0.003	199 (44.9)	184 (41.6)	0.36
Smoking history							
Non-smoker	396 (44.7)	192 (43.3)	204 (46.2)	0.009	203 (45.8)	193 (43.7)	0.07
Ex-smoker	313 (35.4)	145 (32.7)	168 (38.0)		142 (32.1)	171 (38.7)	
Current smoker	176 (19.9)	106 (23.9)	70 (15.8)		98 (22.1)	78 (17.6)	
Atrial fibrillation	14 (1.6)	6 (1.4)	8 (1.8)	0.78	8 (1.8)	6 (1.4)	0.79
Cerebrovascular disease	38 (4.3)	18 (4.1)	20 (4.5)	0.87	16 (3.6)	22 (5.0)	0.40
PVD	17 (1.9)	8 (1.8)	9 (2.0)	1.00	7 (1.6)	10 (2.3)	0.63
Chest pain type							
Non-anginal	303 (34.2)	179 (40.4)	124 (28.1)	<0.001	183 (41.3)	120 (27.1)	<0.001
Atypical angina	211 (23.8)	119 (26.9)	92 (20.8)		108 (24.4)	103 (23.3)	
Typical angina	371 (41.9)	145 (32.7)	226 (51.1)		152 (34.3)	219 (49.5)	
Systolic blood pressure (mm Hg)	140.0 (128.0–154.0)	138.0 (124.0–151.0)	140.0 (130.0–156.8)	<0.001	140.0 (128.0–152.0)	140.0 (128.0–155.0)	0.30
Diastolic blood pressure (mm Hg)	81.0 (76.0–90.0)	80.0 (75.0–90.0)	82.0 (76.0–90.0)	0.04	82.0 (77.0–90.0)	80.0 (75.0–90.0)	0.12
BMI (kg/m^2^)	28.7 (25.9–32.6)	28.6 (25.5–32.3)	29.0 (26.2–32.9)	0.13	29.1 (26.3–33.3)	28.2 (25.5–32.2)	0.02
Antiplatelet	448 (50.6)	189 (42.7)	259 (58.6)	<0.001	183 (41.3)	265 (60.0)	<0.001
Statin	393 (44.4)	171 (38.6)	222 (50.2)	0.001	158 (35.7)	235 (53.2)	<0.001
ACE inhibitor	123 (13.9)	40 (9.0)	83 (18.8)	<0.001	48 (10.8)	75 (17.0)	0.01
Beta-blocker	202 (22.8)	84 (19.0)	118 (26.7)	0.008	72 (16.3)	130 (29.4)	<0.001
ASSIGN score	16.0 (10.0–23.0)	13.0 (8.0–21.0)	18.0 (13.0–26.0)	<0.001	14.0 (9.0–22.0)	17.0 (12.0–25.0)	<0.001
Creatinine (µmol/L)	91.1 (76.7–109.5)	88.6 (74.4–106.3)	93.5 (79.6–111.7)	<0.001	91.4 (77.4–111.4)	90.6 (76.5–108.2)	0.3
hs-cTnI (ng/L)	1.3 (0.9–2.1)	0.9 (0.7–1.1)	2.1 (1.6–3.2)	<0.001	1.2 (0.8–1.8)	1.5 (1.0–2.4)	<0.001
BNP (ng/L)	29.1 (14.2–54.0)	24.9 (11.2–44.1)	34.9 (19.2–67.1)	<0.001	14.2 (7.8–21.7)	54.1 (39.6–83.8)	<0.001
CAD							
Normal	309 (34.9)	204 (46.0)	105 (23.8)	<0.001	178 (40.2)	131 (29.6)	<0.001
Mild (<50%)	175 (19.8)	88 (19.9)	87 (19.7)		94 (21.2)	81 (18.3)	
Moderate (50%–70%)	159 (18.0)	77 (17.4)	82 (18.6)		76 (17.2)	83 (18.8)	
Obstructive (>70%)	242 (27.3)	74 (16.7)	168 (38.0)		95 (21.4)	147 (33.3)	
CT-adapted Leaman score	4.0 (0.0–10.9)	1.5 (0.0–6.9)	7.0 (0.9–14.4)	<0.001	3.1 (0.0–8.7)	5.5 (0.0–12.2)	<0.001
Calcium score (Agatston units)	30.0 (0.0–269.0)	3.0 (0.0–95.0)	103.5 (1.2–546.5)	<0.001	11.0 (0.0–136.5)	63.5 (0.0–430.2)	<0.001
Indexed left ventricular volume (mL/m^2^)	63.2 (56.6–69.5)	63.2 (57.2–69.6)	62.5 (55.8–69.3)	0.30	62.6 (56.2–69.7)	63.2 (56.9–69.4)	0.72
Indexed left ventricular mass (g/m^2^)	71.7 (62.6–81.5)	67.6 (59.6–76.8)	75.7 (67.6–84.4)	<0.001	72.6 (63.4–81.7)	70.8 (61.6–81.2)	0.17
Left ventricular hypertrophy	404 (45.6)	174 (39.3)	230 (52.0)	<0.001	203 (45.8)	201 (45.5)	0.97

Continuous variables are presented as median (IQR).

Categorical variables are presented as n (%).

BMI, body mass index; BNP, B-type natriuretic peptide; CAD, coronary artery disease; CTCA, CT coronary angiography; hs-cTnI, high-sensitivity cardiac troponin I; PVD, peripheral vascular disease.

### Coronary artery disease

Coronary CT angiography demonstrated normal coronary arteries in 309 (35%) patients, while 401 (45%) patients had stenoses of at least 50% (table 1). The median calcium score was 30 (0.00–269.0) Agatston units. The median CT-adapted Leaman score was 4.0 (0.00–10.9), and 406 (46%) patients had high-risk coronary artery disease (CT-adapted Leaman score >5). In 576 patients with evidence of any coronary artery disease on CT, the median calcium score was 143.5 (31.0–530.5) Agatston units, while the median CT-adapted Leaman score was 8.3 (4.4–14.2).

### Left ventricular mass and volume

The median indexed left ventricular mass was 78.1 (71.1–86.1) g/m^2^ for men and 62.6 (56.7–69.9) g/m^2^ for women. Nearly half (n=404, 46%) of patients met sex-specific CT criteria for left ventricular hypertrophy. The median indexed left ventricular volume was 64.6 (56.6–71.3) mL/m^2^ for men and 62.1 (56.5–68.0) mL/m^2^ for women.

### Determinants of plasma cardiac biomarkers

**Figure 2:**
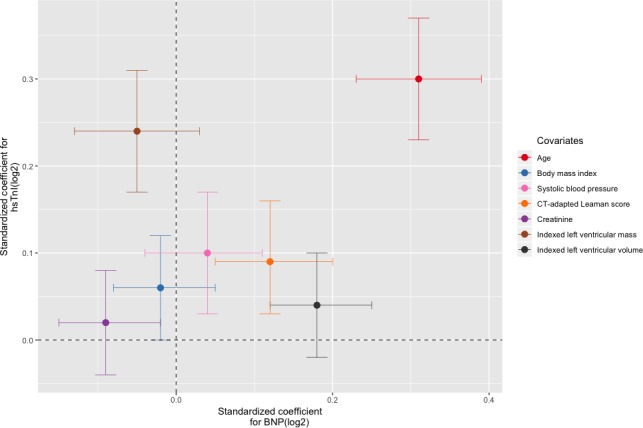
Multivariable logistic regression analysis for cardiac biomarkers. Multivariable logistic regression and relative contribution from selected covariates to biomarker concentrations. Log-transformed continuous covariates from the final multivariable models for high-sensitivity troponin I (hsTnI) and B-type natriuretic peptide (BNP) were selected and scaled ((x – mean)/SD) in order to present each variable’s relative contribution to the final model. 95% CIs are provided for both troponin (y axis) and BNP (x axis).

Univariable and sequential multivariable linear regression were performed using the prespecified variables for troponin (table 2) and BNP (table 3). There was an association between troponin and BNP measurements on univariable linear regression for both men and women (figure 1). For the final multivariable models, all prespecified covariates for troponin (r^2^=0.30, p<0.001) and BNP (r^2^=0.19, p<0.001) were included. Increasing age and atherosclerotic burden as assessed by CT-adapted Leaman score were independent predictors for increased troponin and BNP concentrations. Male sex and indexed left ventricular mass were predictors of increased troponin, in addition to hypertension, systolic blood pressure and body mass index, but not BNP. In contrast, female sex and indexed left ventricular volume were predictors of increased BNP but not troponin. Of note, creatinine was a univariable predictor of increased troponin, but the association no longer remained after adjustment for other variables. Conversely, creatinine was a multivariable predictor of lower BNP. The biomarker-specific nature of these associations was confirmed on multivariable analysis of variance testing of the full model where both biomarkers were included as dependent variables (bivariate multivariable regression). Furthermore, after standardising continuous variables, indexed left ventricular mass and volume provided the largest relative contribution to the explained variance within the troponin and BNP multivariable models, respectively, apart from age, which demonstrated the highest coefficient for both models (figure 2). Finally, to assess the potential effect of data imputation, complete case analysis was performed (n=495). Pooled model parameters were also determined (online supplementary tables 3 and 4). The multivariable models were largely unchanged. The direction and magnitude for the associations between age, gender, structural CT parameters, creatinine and both biomarkers were similar, although with expected variance in SEs.

**Table 2:** Univariable and multivariable associations for high-sensitivity cardiac troponin I concentration

	Univariable regression analysis			Multivariable regression analysis*: r^2^=0.30		
	Coefficient	95% CI	P value	Coefficient	95% CI	P value
Age (per 10 years)	0.37	0.30 to 0.44	<0.001	0.33	0.25 to 0.41	<0.001
Male	0.60	0.47 to 0.73	<0.001	0.25	0.10 to 0.40	0.002
Hypertension	0.49	0.35 to 0.63	<0.001	0.17	0.04 to 0.30	0.011
Hyperlipidaemia	0.29	0.15 to 0.43	<0.001			
Diabetes	0.19	−0.04 to 0.41	0.11			
Documented CAD	0.40	0.16 to 0.64	0.001			
Family history of CAD	−0.23	−0.37 to −0.10	0.001			
Ex-smoker	0.03	−0.12 to 0.19	0.68			
Current smoker	−0.15	−0.34 to 0.03	0.10			
BMI (log_2_)	0.15	−0.11 to 0.41	0.27	0.24	0.00 to 0.47	0.048
Systolic blood pressure (log_2_)	1.16	0.83 to 1.49	<0.001	0.52	0.17 to 0.87	0.004
Diastolic blood pressure (log_2_)	0.49	0.16 to 0.82	0.004			
CT-adapted Leaman score (log_2_)	0.19	0.16 to 0.23	<0.001	0.06	0.02 to 0.10	0.007
Indexed left ventricular mass (log_2_)	1.37	1.13 to 1.61	<0.001	0.92	0.64 to 1.20	<0.001
Indexed left ventricular volume (log_2_)	0.01	−0.27 to 0.28	0.96			
Creatinine (log_2_)	0.41	0.22 to 0.61	<0.001			

*All covariates were included in the final multivariable linear regression model; only those with p<0.05 are presented. Full modelling is presented in online supplementary table 1.

BMI, body mass index; CAD, coronary artery disease.

**Table 3:** Univariable and multivariable associations for B-type natriuretic peptide

	Univariable regression analysis			Multivariable regression analysis*: r^2^=0.18		
	Coefficient	95% CI	P value	Coefficient	95% CI	P value
Age (per 10 years)	0.6	0.49 to 0.71	<0.001	0.54	0.41 to 0.67	<0.001
Male	−0.32	−0.54 to −0.10	0.005	−0.33	−0.60 to −0.06	0.016
Hypertension	0.31	0.09 to 0.54	0.007			
Hyperlipidaemia	0.55	0.33 to 0.77	<0.001			
Diabetes	−0.22	−0.58 to 0.14	0.23			
Documented CAD	0.74	0.35 to 1.12	<0.001	0.46	0.09 to 0.84	0.016
Family history of CAD	−0.07	−0.30 to 0.15	0.52			
Ex-smoker	0.22	−0.03 to 0.47	0.082			
Current smoker	−0.24	−0.54 to 0.05	0.11			
BMI (log_2_)	−0.45	−0.87 to −0.03	0.035			
Systolic blood pressure (log_2_)	0.61	0.06 to 1.16	0.028			
Diastolic blood pressure (log_2_)	−0.36	−0.90 to 0.18	0.19			
CT-adapted Leaman score (log_2_)	0.2	0.13 to 0.26	<0.001	0.12	0.05 to 0.19	0.001
Indexed left ventricular mass (log_2_)	−0.3	−0.71 to 0.11	0.15			
Indexed left ventricular volume (log_2_)	0.3	−0.15 to 0.74	0.19	1.24	0.79 to 1.69	<0.001
Creatinine (log_2_)	−0.39	−0.71 to −0.06	0.019	−0.42	−0.74 to −0.11	0.008

*All covariates were included in the final multivariable linear regression model; only those with p<0.05 are presented. Full modelling is presented in online supplementary table 2.

BMI, body mass index; CAD, coronary artery disease.

## Discussion

In this analysis from the SCOT-HEART trial, we describe clinical determinants of plasma high-sensitivity cardiac troponin I and BNP concentrations in a cohort of patients with suspected stable angina. We demonstrate the ability of cardiac CT to identify and characterise three distinct determinants of these biomarker concentrations: atherosclerotic burden, left ventricular mass and left ventricular volume. This has particular clinical relevance as troponin and BNP are powerful predictors of cardiovascular risk. These quantifiable imaging measures offer potentially modifiable targets for intervention.

It is imperative that patient-specific mechanisms for biomarker concentrations are understood. Are they a marker of an active, modifiable disease process, or simply sentinels for generalised, systemic inflammation and elevated cardiovascular risk? Without this understanding, targeted interventions cannot be applied, thereby diminishing the clinical benefits of additional prognostic information. The importance of a mechanistic approach is demonstrated by the success of canakinumab, which specifically targets inflammation as a driver of cardiovascular events.^23^ Importantly, a number of therapies have already been demonstrated to address the processes we identified as contributing to increased biomarker concentrations. This provides a strong basis for using cardiac imaging when investigating the cause of these increased biomarker concentrations.

Our findings suggest that cardiac CT can achieve some of the necessary characterisation of biomarker determinants in stable patients. Obstructive coronary artery disease correlates with troponin concentrations in patients with stable angina.^24^ Here, we applied the CT-adapted Leaman score and demonstrate that, in addition to other clinical characteristics, this association persists after adjustment for left ventricular mass and volume. Although atherosclerotic disease is a chronic inflammatory process, changes within the vasculature are distinct from structural processes; cardiac CT facilitates the interrogation of both these aspects. Left ventricular mass measured at end-diastole using other modalities has been shown to correlate with elevations in cardiac biomarkers.^25^ Our study confirms a comparable relationship between troponin and mid-diastolic assessment of left ventricular mass on gated CT. Additionally, BNP concentrations are elevated in heart failure or conditions with increased wall stress leading to left ventricular hypertrophy.^9^ However, we have demonstrated that after adjustment, left ventricular volume remains an independent determinant of BNP in stable patients without evidence of heart failure, whereas mass does not. The ability to perform these measurements with prospective gating is highly relevant in current practice. Of note, although we were unable to compare mid-diastolic left ventricular mass or volume directly with echocardiography or cardiac MRI, excellent correlation with other cardiac phases has been demonstrated previously.^15 17 18^

Our study is consistent with prior reports demonstrating an association between plasma biomarker concentrations and several clinical factors. Advanced age and male gender are both associated with increased troponin concentrations.^6 8^ There has been considerable research into determining age-specific and sex-specific 99th centile thresholds for each assay. Although posited explanations include a higher atherosclerotic burden in the elderly and increased myocardial mass in men, we have shown that age and male gender remain predictors of higher troponin concentrations even after adjusting for these variables. Likewise, we also describe the association between female sex and increased BNP which persisted after adjustment for confounders such as age, atherosclerotic burden and left ventricular mass and volume—variables that are not reported in previous studies. This demonstrates the utility of cardiac CT in delineating how coronary and structural parameters contribute to BNP elevation.^3^ The precise mechanisms for these independent gender differences are unclear. Hormonal differences are likely to contribute. Oestrogen has an attenuating effect on atherosclerosis and left ventricular hypertrophy,^26^ while testosterone suppresses natriuretic peptide production, resulting in lower concentrations.^27^ Although the mechanisms are uncertain, our findings support the application of sex-specific reference ranges, and furthermore point towards the need for age-specific cut-offs. Universal thresholds may misclassify patients, potentially leading to inappropriate therapies.

In contrast to prior reports that elevated troponin concentrations are more common in outpatients with chronic kidney disease,^28 29^ we found that a higher creatinine was not an independent predictor of increased troponin. In contrast, creatinine was associated with a lower BNP concentration after multivariable adjustment. Although unexpected, it should be noted BNP has been reported as less sensitive to changes in renal function than N-terminal proBNP, while increased concentrations are typically seen in patients with advanced renal impairment that would have been ineligible for the SCOT-HEART trial.^29^ Indeed, there were relatively few patients in this cohort with renal impairment. Furthermore, as discussed below, there is a degree of variability in BNP assays that is driven by several factors. Small changes in serum creatinine may also be caused by factors which we did not control for, such as certain medications. Nevertheless, our findings suggest that in the context of preserved or mildly impaired renal function, changes in plasma troponin I and BNP concentrations are due to comorbid conditions rather than renal dysfunction.

Our study has several strengths. First, we applied a troponin assay with exceptional analytical characteristics, enabling us to detect circulating troponin in 99.6% of the study population and to determine troponin concentrations accurately in 96.8%. Second, we made use of state-of-the-art CT imaging using a 320-slice scanner to quantify coronary atherosclerotic burden and left ventricular mass and volume according to previously validated techniques. Third, as this study was nested within a trial randomising allocation to CT imaging, we have minimised case ascertainment bias that may arise when imaging decisions are dependent on clinical suspicion of coronary disease. Finally, we achieved detailed and accurate phenotypic characterisation of participants.

There are several limitations. First, key exclusion criteria for the study included age >75 years or severe renal impairment (estimated glomerular filtration rate <30 mL/min/1.73 m^2^); the determinants of biomarker concentrations in these contexts therefore remain uncertain. Nevertheless, SCOT-HEART was pragmatic in design, enrolling a cohort broadly representative of real-world populations with suspected stable angina. Second, without serial plasma samples, we cannot be certain that biomarker values reflect true baseline concentrations. However, participants were recruited in a stable outpatient setting and we excluded individuals with troponin concentrations above the 99th centile and BNP >400 ng/L,^13^ thereby minimising the possibility of including patients with acute myocardial injury or heart failure. Third, there is some biological, genetic and analytical variability in biomarker concentrations. Current BNP assays measure a mixture of peptides, including degradation products and proBNP, while degradation in frozen BNP samples over time is well described. These limitations are common to current assays, including N-terminal proBNP assays. Furthermore, the clinical characteristics associated with troponin and BNP that we describe are largely consistent with existing data, supporting the validity of our analysis, while BNP degradation appears to be more relevant at higher concentrations.^30^ Fourth, although the assessment of left ventricular mass and volume using cardiac CT is an accepted and accurate method, mid-diastolic assessment is less widely studied. This may account for the relatively high prevalence of left ventricular hypertrophy as defined by previously published thresholds, although more than one-third of patients had a diagnosis of hypertension. Finally, despite the detailed characterisation of this cohort, substantial unexplained variance in troponin and BNP concentrations remains, highlighting the need for ongoing research to better understand the information that these assays offer.

## Conclusions

Detectable plasma high-sensitivity cardiac troponin I and BNP concentrations are present in almost all patients with suspected stable coronary artery disease. These biomarkers are associated with atherosclerotic burden, but measures of underlying left ventricular structure are associated with each biomarker differentially. Cardiac biomarkers provide information regarding patient-specific modifiable targets for therapy, allowing personalisation of treatment that may further help reduce cardiovascular risk.

**Key messages**

**What is already known on this subject?**

- Increased cardiac troponin and B-type natriuretic (BNP) concentrations are associated with greater risk of cardiovascular events in individuals with and without established cardiac disease.
- Plasma concentrations in stable outpatients may be attributable to pathophysiological processes including hypertension, coronary atherosclerosis and left ventricular dysfunction, but clinical determinants are not fully characterised.

**What might this study add?**

- We demonstrate the association between overall plaque burden as assessed by CT coronary angiography (CTCA) and troponin and BNP concentrations.
- Importantly, we describe sex differences and contrasting associations with left ventricular mass and volume.
- We also demonstrate the ability of prospectively gated CTCA performed primarily for coronary artery assessment to provide additional information regarding ventricular mass and volume.

**How might this impact on clinical practice?**

- Although plasma troponin and BNP have prognostic value in stable patients, an understanding of their determinants—and specifically, modifiable determinants—is necessary to add clinical utility.
- With this knowledge, these plasma biomarkers have the potential to act as simple surrogate markers of treatment efficacy for targeted disease-modifying therapies.
