# Supplementary material for: Clinical determinants of plasma cardiac biomarkers in patients with stable chest pain
Source: Heart. 2019 Jun 1;105(22):1748–54. doi: 10.1136/heartjnl-2019-314892 (PMC6855840; doi:10.1136/heartjnl-2019-314892)
Supplement: Supplementary file 1 [file heartjnl-2019-314892supp001.docx]

| **Supplementary Table 1** Multivariable associations for high-sensitivity cardiac troponin I concentration | | | | | | | | | |
| --- | --- | --- | --- | --- | --- | --- | --- | --- | --- |
|  | **Model 1**  **r^2^ = 0.19** | | | **Model 2**  **r^2^ = 0.29** | | | **Model 3**  **r^2^ = 0.30** | | |
|  | **Coefficient** | **95% CI** | **p-value** | **Coefficient** | **95% CI** | **p-value** | **Coefficient** | **95% CI** | **p-value** |
| **Indexed left ventricular mass (log2)** | 1.23 | 0.98 to 1.48 | <0.001 | 1.02 | 0.74 to 1.30 | <0.001 | 0.92 | 0.64 to 1.20 | <0.001 |
| **CT-adapted Leaman score (log2)** | 0.15 | 0.11 to 0.19 | <0.001 | 0.06 | 0.02 to 0.10 | 0.004 | 0.06 | 0.02 to 0.10 | 0.007 |
| **Indexed left ventricular volume (log2)** | -0.24 | -0.51 to 0.02 | 0.069 | 0.12 | -0.13 to 0.38 | 0.35 | 0.17 | -0.09 to 0.43 | 0.19 |
| **Male** |  |  |  | 0.22 | 0.08 to 0.37 | 0.003 | 0.25 | 0.10 to 0.40 | 0.002 |
| **Age per 10 years** |  |  |  | 0.33 | 0.25 to 0.40 | <0.001 | 0.33 | 0.25 to 0.41 | <0.001 |
| **Hypertension** |  |  |  | 0.23 | 0.10 to 0.35 | 0.001 | 0.17 | 0.04 to 0.30 | 0.011 |
| **Hyperlipidaemia** |  |  |  | 0.02 | -0.10 to 0.15 | 0.74 | 0.01 | -0.11 to 0.14 | 0.83 |
| **Diabetes** |  |  |  | 0.03 | -0.16 to 0.22 | 0.77 | 0.01 | -0.19 to 0.20 | 0.93 |
| **Family history of CAD** |  |  |  | -0.09 | -0.21 to 0.03 | 0.16 | -0.08 | -0.20 to 0.04 | 0.19 |
| **Documented CAD** |  |  |  | 0.00 | -0.21 to 0.22 | 0.97 | 0.05 | -0.17 to 0.26 | 0.66 |
| **Ex-smoker** |  |  |  | -0.09 | -0.26 to 0.08 | 0.29 | -0.11 | -0.28 to 0.06 | 0.20 |
| **Current smoker** |  |  |  | 0.05 | -0.11 to 0.21 | 0.57 | 0.02 | -0.14 to 0.18 | 0.80 |
| **Body mass index (log2)** |  |  |  |  |  |  | 0.24 | 0.00 to 0.47 | 0.048 |
| **Systolic blood pressure (log2)** |  |  |  |  |  |  | 0.52 | 0.17 to 0.87 | 0.004 |
| Abbreviations: CAD = coronary artery disease, CI = confidence interval | | | | | | | | | |

| **Supplementary Table 2** Multivariable associations for B-natriuretic peptide concentration | | | | | | | | | |
| --- | --- | --- | --- | --- | --- | --- | --- | --- | --- |
|  | **Model 1**  **r^2^ = 0.06** | | | **Model 2**  **r^2^ = 0.18** | | | **Model 3**  **r^2^ = 0.19** | | |
|  | **Coefficient** | **95% CI** | **p-value** | **Coefficient** | **95% CI** | **p-value** | **Coefficient** | **95% CI** | **p-value** |
| **Indexed left ventricular mass (log2)** | -0.84 | -1.27 to -0.41 | <0.001 | -0.31 | -0.79 to 0.18 | 0.21 | -0.30 | -0.80 to 0.19 | 0.23 |
| **CT-adapted Leaman score (log2)** | 0.24 | 0.18 to 0.31 | <0.001 | 0.12 | 0.05 to 0.19 | 0.001 | 0.12 | 0.05 to 0.19 | 0.001 |
| **Indexed left ventricular volume (log2)** | 0.78 | 0.32 to 1.24 | 0.001 | 1.27 | 0.82 to 1.72 | <0.001 | 1.24 | 0.79 to 1.69 | <0.001 |
| **Male** |  |  |  | -0.42 | -0.68 to -0.16 | 0.002 | -0.33 | -0.60 to -0.06 | 0.016 |
| **Age per 10 years** |  |  |  | 0.56 | 0.43 to 0.69 | <0.001 | 0.54 | 0.41 to 0.67 | <0.001 |
| **Hypertension** |  |  |  | 0.08 | -0.14 to 0.30 | 0.49 | 0.11 | -0.12 to 0.34 | 0.34 |
| **Hyperlipidaemia** |  |  |  | 0.18 | -0.04 to 0.40 | 0.11 | 0.17 | -0.05 to 0.39 | 0.12 |
| **Diabetes** |  |  |  | -0.28 | -0.62 to 0.05 | 0.099 | -0.27 | -0.62 to 0.07 | 0.12 |
| **Family history of CAD** |  |  |  | 0.09 | -0.12 to 0.30 | 0.41 | 0.09 | -0.12 to 0.30 | 0.38 |
| **Documented CAD** |  |  |  | 0.42 | 0.05 to 0.79 | 0.026 | 0.46 | 0.09 to 0.84 | 0.016 |
| **Ex-smoker** |  |  |  | 0.14 | -0.15 to 0.43 | 0.35 | 0.18 | -0.11 to 0.48 | 0.23 |
| **Current smoker** |  |  |  | 0.10 | -0.18 to 0.38 | 0.50 | 0.12 | -0.16 to 0.40 | 0.39 |
| **Body mass index (log2)** |  |  |  |  |  |  | -0.12 | -0.53 to 0.29 | 0.57 |
| **Systolic blood pressure (log2)** |  |  |  |  |  |  | 0.32 | -0.29 to 0.94 | 0.31 |
| Abbreviations: CAD = coronary artery disease, CI = confidence interval | | | | | | | | | |

**Supplementary Tables 3 and 4 – Multiple imputation models**

Multiple imputation was performed by chained equations (MICE). We included recorded clinical (demographics, past medical history, systolic and diastolic blood pressure, height and weight, and smoking status), biochemical (creatinine concentration), and CT-derived data (LV volumes, LV mass, coronary disease burden) from all participants included in the analysis population. Each variable was regressed on all other variables with 20 iterations performed. Imputations for continuous variables were performed using predictive mean matching using the five nearest neighbours to the prediction as a set to draw from whilst for categorical or ordinal variables we applied logistic regression (binomial and polytomous respectively). We selected the first imputed result for the primary analysis. A sensitivity analysis was also performed with respect to the full multivariable models specified for troponin and BNP, retaining all imputed data and applying Rubin’s rules to determine pooled estimates for the complete data model. These are presented below. Variables with a p-value <0.05 are presented.

| **Supplementary Table 3** Pooled multivariable imputation model for troponin | | | | | | |
| --- | --- | --- | --- | --- | --- | --- |
| **Troponin model** | **Unpooled** | | | **Pooled** | | |
|  | **Coefficient** | **SE** | **p-value** | **Coefficient** | **SE** | **p-value** |
| Age (per 10 years) | 0.33 | 0.04 | <0.001 | 0.36 | 0.04 | <0.001 |
| Male | 0.25 | 0.08 | 0.002 | 0.21 | 0.08 | 0.01 |
| Hypertension | 0.17 | 0.07 | 0.01 | 0.16 | 0.07 | 0.02 |
| Hyperlipidaemia |  |  |  |  |  |  |
| Diabetes |  |  |  |  |  |  |
| Documented CAD |  |  |  |  |  |  |
| Family history of CAD |  |  |  |  |  |  |
| Ex-smoker |  |  |  |  |  |  |
| Current smoker |  |  |  |  |  |  |
| BMI (log2) | 0.24 | 0.12 | 0.048 | 0.25 | 0.12 | 0.04 |
| Systolic blood pressure (log2) | 0.52 | 0.18 | 0.004 | 0.49 | 0.18 | 0.006 |
| Diastolic blood pressure (log2) |  |  |  |  |  |  |
| CT-adapted Leaman score (log2) | 0.06 | 0.02 | 0.007 | 0.06 | 0.02 | 0.01 |
| Indexed left ventricular mass (log2) | 0.92 | 0.14 | <0.001 | 1.02 | 0.16 | <0.001 |
| Indexed left ventricular volume (log2) | 0.17 | 0.13 | 0.19 | 0.36 | 0.16 | 0.03 |
| Creatinine (log2) |  |  |  |  |  |  |
| Abbreviations: SE = standard error, CAD = coronary artery disease, BMI = body mass index, CT = computed tomography | | | | | | |

| **Supplementary Table 4** Pooled multivariable imputation model for BNP | | | | | | |
| --- | --- | --- | --- | --- | --- | --- |
| **BNP model** | **Unpooled** | | | **Pooled** | | |
|  | **Coefficient** | **SE** | **p-value** | **Coefficient** | **SE** | **p-value** |
| Age (per 10 years) | 0.54 | 0.07 | <0.001 | 0.59 | 0.07 | <0.001 |
| Male | -0.33 | 0.14 | 0.016 | -0.26 | 0.14 | 0.07 |
| Hypertension |  |  |  |  |  |  |
| Hyperlipidaemia |  |  |  |  |  |  |
| Diabetes |  |  |  |  |  |  |
| Documented CAD | 0.46 | 0.19 | 0.016 | 0.44 | 0.20 | 0.03 |
| Family history of CAD |  |  |  |  |  |  |
| Ex-smoker |  |  |  |  |  |  |
| Current smoker |  |  |  |  |  |  |
| BMI (log2) |  |  |  |  |  |  |
| Systolic blood pressure (log2) |  |  |  |  |  |  |
| Diastolic blood pressure (log2) |  |  |  |  |  |  |
| CT-adapted Leaman score (log2) | 0.12 | 0.04 | 0.001 | 0.11 | 0.04 | 0.003 |
| Indexed left ventricular mass (log2) |  |  |  |  |  |  |
| Indexed left ventricular volume (log2) | 1.24 | 0.23 | <0.001 | 1.35 | 0.26 | <0.001 |
| Creatinine (log2) | -0.42 | 0.16 | 0.008 | -0.62 | 0.20 | 0.002 |
| Abbreviations: BNP = B-type natriuretic peptide , SE = standard error, CAD = coronary artery disease, BMI = body mass index, CT = computed tomography | | | | | | |
